# Binucleated human bone marrow-derived mesenchymal cells can be formed during neural-like differentiation with independence of any cell fusion events

**DOI:** 10.1038/s41598-022-24996-8

**Published:** 2022-11-30

**Authors:** Carlos Bueno, Miguel Blanquer, David García-Bernal, Salvador Martínez, José M. Moraleda

**Affiliations:** 1 Medicine Department and Hematopoietic Transplant and Cellular Therapy Unit, Institute of Biomedical Research (IMIB), Faculty of Medicine, University of Murcia, 30120 Murcia, Spain; 2 Biochemistry, Molecular Biology and Immunology Department, Faculty of Medicine, University of Murcia, 30100 Murcia, Spain; 3 Instituto de Neurociencias de Alicante (UMH-CSIC), Universidad Miguel Hernandez, 03550 San Juan, Alicante, Spain

**Keywords:** Mesenchymal stem cells, Adult neurogenesis, Stem-cell differentiation

## Abstract

Although it has been reported that bone marrow-derived cells (BMDCs) can transdifferentiate into neural cells, the findings are considered unlikely. It has been argued that the rapid neural transdifferentiation of BMDCs reported in culture studies is actually due to cytotoxic changes induced by the media. While transplantation studies indicated that BMDCs can form new neurons, it remains unclear whether the underlying mechanism is transdifferentiation or BMDCs-derived cell fusion with the existing neuronal cells. Cell fusion has been put forward to explain the presence of gene-marked binucleated neurons after gene-marked BMDCs transplantation. In the present study, we demostrated that human BMDCs can rapidly adopt a neural-like morphology through active neurite extension and binucleated human BMDCs can form with independence of any cell fusion events. We also showed that BMDCs neural-like differentiation involves the formation of intermediate cells which can then redifferentiate into neural-like cells, redifferentiate back to the mesenchymal fate or even repeatedly switch lineages without cell division. Furthermore, we have discovered that nuclei from intermediate cells rapidly move within the cell, adopting different morphologies and even forming binucleated cells. Therefore, our results provide a stronger basis for rejecting the idea that BMDCs neural transdifferentiation is merely an artefact.

## Introduction

The fate of adult cells has been thought to be restricted to their tissues of origin^1^. However, recent studies have indicated that certain mammalian adults cells may be more plastic than we previously thought in that they maintain the ability for multilineage cell differentiation and may turn into cells of unrelated lineages, a phenomenon known as adult cell plasticity^2–5^. The first suggestion that adult cells switch into another cell type of an unrelated tissue came from studies of whole bone marrow transplantation in humans and animal models. These studies suggested that bone marrow-derived cells (BMDCs) could enter the brain and transdifferentiate into cells with a neuronal-specific phenotype^6–8^. Bone marrow contains two prototypical stem cell population: haematopoietic stem cells (HSCs) and mesenchymal stromal cells (MSCs). Whereas HSCs give rise to the various blood cells, bone marrow-derived MSCs can differentiate into mesodermal lineage cells such as osteocytes, chondrocytes, and adipocytes^9^. Nevertheless, several reports have indicated that BMDCs and MSCs isolated from different adult tissues can also transdifferentiate into neural cells, both in vitro^10–18^ and in vivo^6–8,19–23^. However, the findings and their interpretation have been challenged^24,25^. The main argument against these observations in culture studies is that MSCs rapidly adopt neural-like morphologies through retraction of the cytoplasm, rather than by active neurite extension^24,26–28^. While transplantation studies have indicated that BMDCs can contribute to the neuronal architecture of the nervous system, including Purkinje cells within the cerebellum^6–8,21–23^, the possibility of BMDCs transdifferenting into neural cells is considered unlikely, and the more accepted explanation that donor BMDCs fuse with host neurons^25^. Cell fusion has been put forward to explain the presence of gene-marked binucleated Purkinje neurons after gene-marked bone marrow-derived cell transplantation^29,30^. The actual occurrence of neuronal transdifferentiation of BMDCs and MSCs is currently much debated, but would have immense clinical potential in cell replacement therapy of neurodegenerative diseases^31^.

In previous publications, we showed that MSCs isolated from adult human tissues (hMSCs) can differentiate into neural-like cells, both in vitro and in vivo^32–34^. In vivo, hMSCs-derived neural-like cells survived, migrated and expressed neural markers after being grafted to the adult mouse brain^32^. Importantly, the hMSCs-derived neural-like cells located in the neural stem cell niches show neural stem morphology. In vitro studies have shown that hMSCs can differentiate into neural-like cells without passing through a mitotic stage and that they shrank dramatically and changed their morphology to that of neural-like cells through active neurite extension^33,34^. The hMSCs-derived neural-like cells expressed neural-specific gene products and produced neurites that show growth cone formations at their tip, with a similar spatial organisation as that described in neurons. Futhermore, we noted that hMSCs-derived neural-like cells comprised multiple branched dendrite-like processes with micron-sized membrane protrusions, making these resembles the typical dendritic spine structures described in neurons. In addition, hMSCs-derived neural-like cells also displayed different types of axonal branch-like structures that are very similar to those described in neurons.

It is important to noted that nuclear remodelling occurred during in vitro neural-like differentiation from hMSCs. We discovered that many hMSCs exhibit unusual nuclear structures and even possess two nuclei. In the present study, we examined the sequence of biological events during neural-like differentiation of human bone marrow-derived MSCs (hBM-MSCs) by live-cell nucleus fluorescence labelling and time-lapse microscopy to determine whether the binucleation events observed during neural-like differentiation from hMSCs are due to cell division or cell fusion events.

## Results

### Morphological changes in hBM-MSCs during neural-like differentiation

Under proliferation conditions, hBM-MSCs displayed a flat, fibroblast-like morphology with little evidence of refractility (Fig. S1a). These cells did not express hematopoietic lineage markers such as CD14, CD20, CD34 and CD45, and were positive for CD73, CD90, CD105, thereby demonstrating a characteristic immunophenotype of hMSC (Fig. S1b).

**Figure 1:**
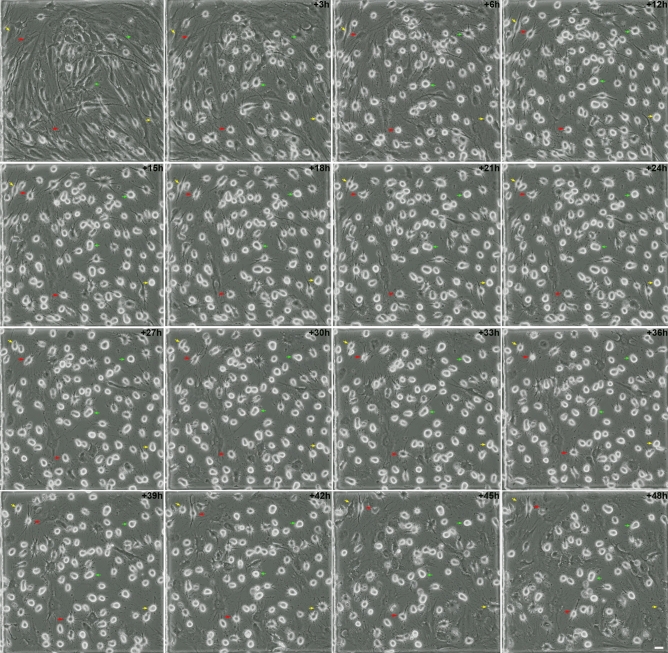
Morphological changes in hBM-MSC cultures during neural-like differentiation. Time-lapse imaging revealed that, following neural induction, hBM-MSCs rapidly reshaped from a flat to a spherical morphology (intermediate cells). Subsequently, we observed that hBM-MSC-derived intermediate cells can preserve their spherical shape for several days (green arrows), change to that of neural-like cells through active neurite extension (red arrows) or revert back to the mesenchymal morphology (yellow arrows). Scale bar: 25 µm.

We examined the sequence of biological events during neural-like differentiation of histone H2B-GFP transfected hBM-MSCs by time-lapse microscopy. Time-lapse imaging revealed that, after neural induction, hBM-MSCs can rapidly reshape from a flat to a spherical morphology (Fig. 1). The mayority of hBM-MSCs assumed a spherical morphology (hBM-MSC-derived intermediate cells) within 12 h of exposure to neural induction medium (Fig. S2). The hBM-MSC-derived intermediate cells are attached to the cell culture dish and exhibited highly refractile rounded cell bodies.

Subsequently, we observed that hBM-MSC-derived intermediate cells can maintain the spherical shape (Fig. 1, green arrows and Supplementary Video S1) or assume new morphologies; intermediate cells can change to a morphology similar to that of neural-like cells through active neurite extension (Fig. 1, red arrows and Supplementary Video S1) or they can revert back to the mesenchymal morphology (Fig. 1, yellow arrows and Supplementary Video S1). The hBM-MSCs did not differentiate at the same time or rate, so the cell culture simultaneously contained hBM-MSCs at different stages of neural-like differentiation. Importantly, there was no cell proliferation or cell fusion through neural-like differentiation from hBM-MSCs (Fig. 1 and Supplementary Video S1). These results confirm our previous findings^33,34^ and lend further support to the notion that MSCs transdifferentiate towards a neural lineage through a dedifferentiation step followed by re-differentiation to neural phenotypes, thus definitively confirming that the rapid acquisition of a neural-like morphology during hMSC neural-like differentiation is via a differentiation trait rather than merely an artefact.

**Figure 2:**
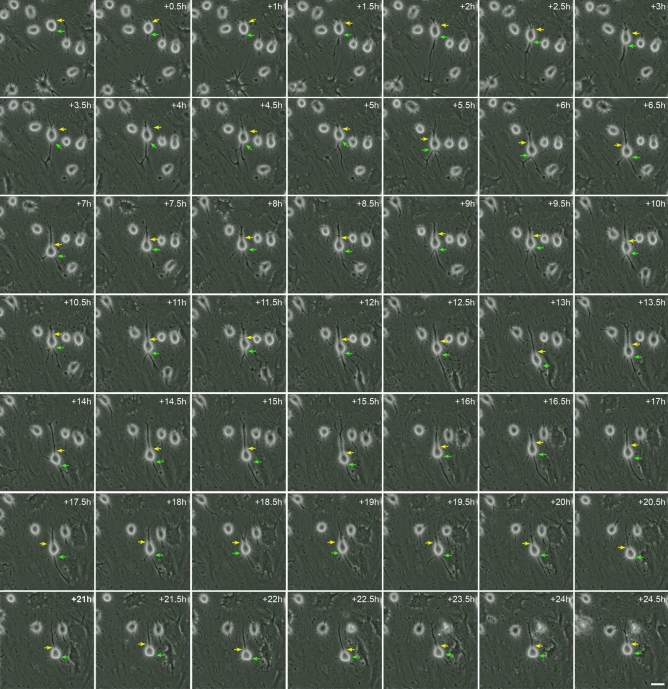
Neuronal-like polarisation of hBM-MSC-derived intermediate cells. Time-lapse imaging revealed the growth of new neurites from the body of round cells (intermediate cells) that which gradually adopted a complex morphology, acquiring dendrite-like (green arrows) and axon-like domains (yellow arrows). There was no observation of major transient cellular protrusion as hBM-MSC-derived intermediate cells gradually acquired a neural-like morphology. Scale bar: 25 µm.

As noted above, hBM-MSC-derived intermediate cells can even preserve their spherical morphology for days without assuming new fates. However, it is important to note that cellular protrusions appeared, moved and disappeared from the surface of hBM-MSC-derived intermediate cells during this dedifferentiation-like stage (Fig. S3 and Supplementary Video S2). Contrastingly, we also found that hBM-MSC-derived intermediate cells can adopt a neural-like morphology via active neurite extension. (Fig. 2 and Supplementary Video S3). New neurites grew from the body of some round cells, which gradually adopted a more complex morphology, by acquiring dendrite-like (Fig. 2, green arrows) and axon-like domains (Fig. 2, yellow arrows). Time-lapse imaging also revealed that the hBM-MSC-derived neural-like cells create connections via extensions of their neurites (Fig. S4 and Supplementary Video S4). These results confirm our previous findings^33,34^ and lend further support to the notion that hMSCs-derived neural-like cells establish their polarity in a very similar way to that previously reported for cultured rodent neurons^33^.

**Figure 3:**
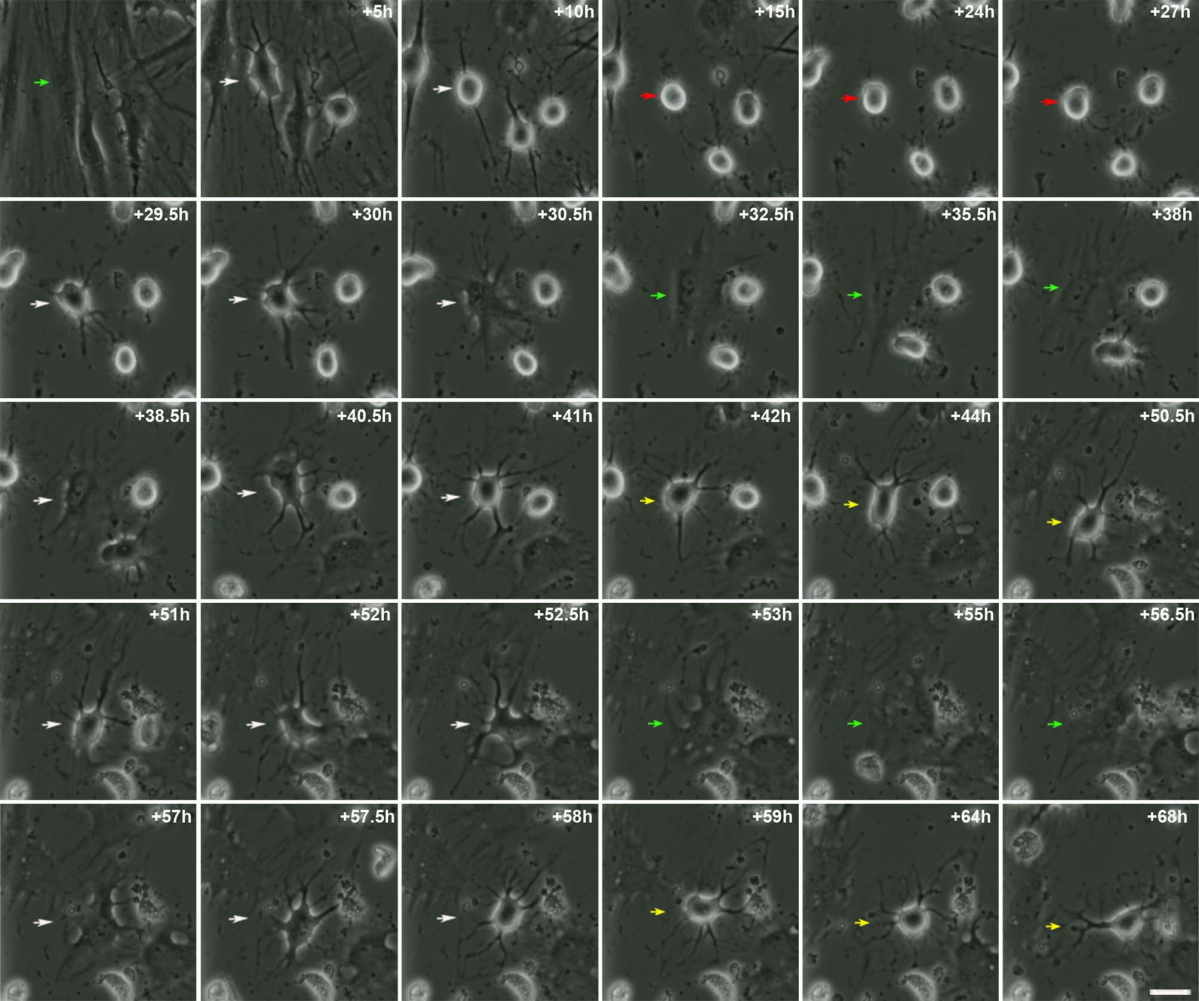
hBMSCs can repeatedly switch lineages. Time-lapse imaging showed that hBM-MSCs can also rapidly switch lineages without cell division. Mesenchymal morphology (green arrows); switching lineages (white arrows); intermediate morphology (red arrows); neural-like morphology (yellow arrows). Scale bar: 25 µm.

Finally, hBM-MSC-derived intermediate cells and neural-like cells could also re-differentiate back to the mesenchymal morphology (Figs. 1 and 2 and Supplementary Videos S1 and S2). Surprisingly, hBM-MSCs could also rapidly and repeatedly switch lineages without cell division (Fig. 3 and Supplementary Video S5). This finding is consistent with a previous study that report Schwann cells can undergo multiple cycles of differentiation and dedifferentiation without entering the cell cycle^35^.

### Nuclear remodelling during neural-like differentiation from hBM-MSCs

**Figure 4:**
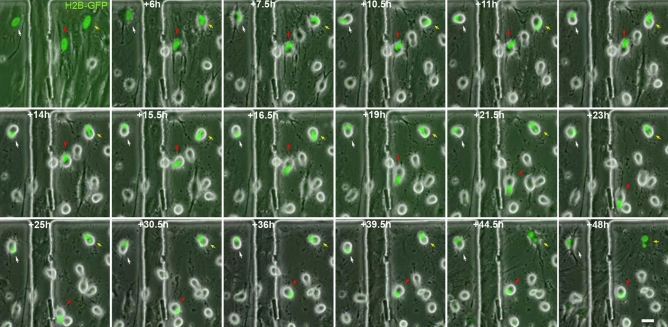
Nucleus remodelling occurs during neural-like differentiation from hBM-MSCs. Time-lapse microscopy evidenced that nuclear remodelling occurred during neural-like differentiation from histone H2B-GFP-expressing hBM-MSCs. Nuclei from hBM-MSC-derived intermediate cells moved within the cell, adopting different morphologies, including finger shaped (red arrows) and kidney shaped (white arrows), and even forming lobed nuclei connected by nucleoplasmic bridges (yellow arrows). Scale bar: 25 µm.

**Figure 5:**
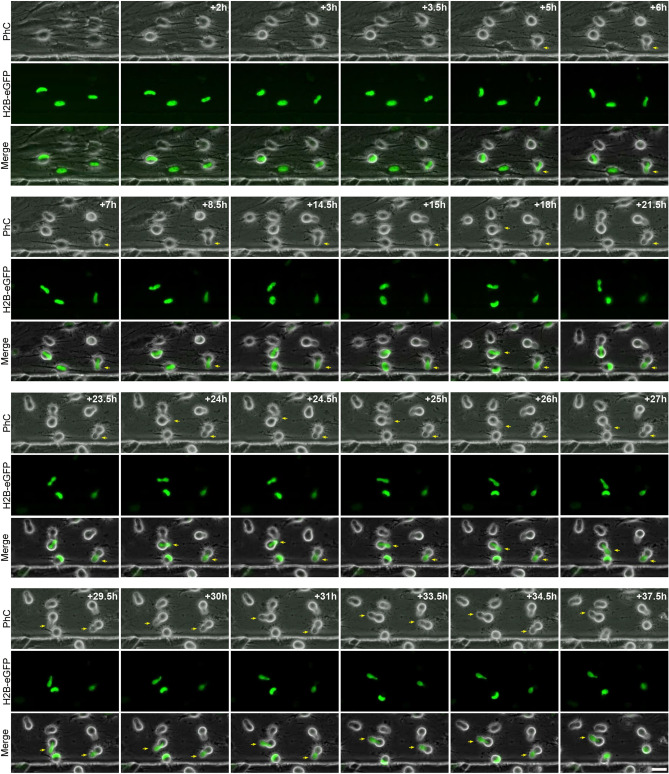
Nuclear movement generated cellular protrusions that appeared and disappeared from the surface of hBM-MSC-derived intermediate cells. Time-lapse microscopy revealed that the cell nucleus of histone H2B-GFP-expressing hBM-MSCs acquired a finger-like shape and moved within the cell, generating the transient cellular protrusions (arrows) on the surface of the hBM-MSC-derived intermediate cells. Scale bar: 25 µm. *PhC* Phase-contrast photomicrographs.

**Figure 6:**
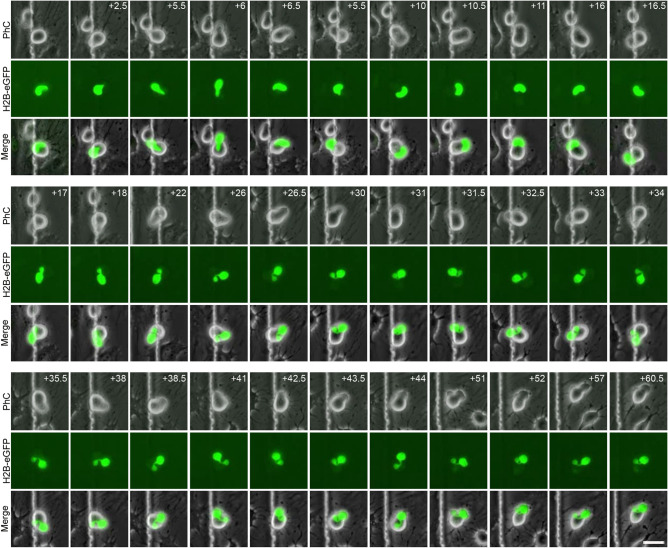
Binucleated hBM-MSCs can form with independence of any cell fusion events. Time-lapse microscopy revealed that the nuclei from histone H2B-GFP-expressing, hBM-MSC-derived intermediate cells can move within the cell, forming lobed nuclei connected by nucleoplasmic bridges. The movement of the lobed nuclei also generated cellular transient protrusions from the surface of hBM-MSC-derived intermediate cells. Scale bar: 25 µm. *PhC* Phase-contrast photomicrographs.

Live-cell nucleus fluorescence labelling and time-lapse microscopy revealed that nuclear remodelling occurred during neural-like differentiation from hBM-MSCs. Nuclei from histone H2B-GFP-expressing, hBM-MSC-derived intermediate cells moved within the cell, adopting different morphologies and positions, and even forming lobed nuclei (Fig. 4 and Supplementary Video S6). Although the cell nuclei switched their morphologies while moving, the nuclear movement primarily produces three different nuclear morphologies and positions. Firstly, the cell nucleus acquired a finger-like shape and moves within the cell, generating the cellular protrusions that appeared and disappeared from the surface of hBM-MSC-derived intermediate cells (Fig. 5 and Supplementary Video S7). Secondly, the nucleus acquired a finger-like shape, before reorienting towards a peripheral position within the cell and acquiring a kidney-like shape. Subsequently, the cell nucleus began to move rapidly around the cell (Fig. S5 and Supplementary Video S8). And thirdly, the nucleus acquired a finger-like shape and moved within the cell to form lobed nuclei connected by nucleoplasmic bridges (Fig. 6 and Supplementary Video S9). The lobed nuclei movement also generated transient cellular protrusions on the surface of hBM-MSC-derived intermediate cells.

**Figure 7:**
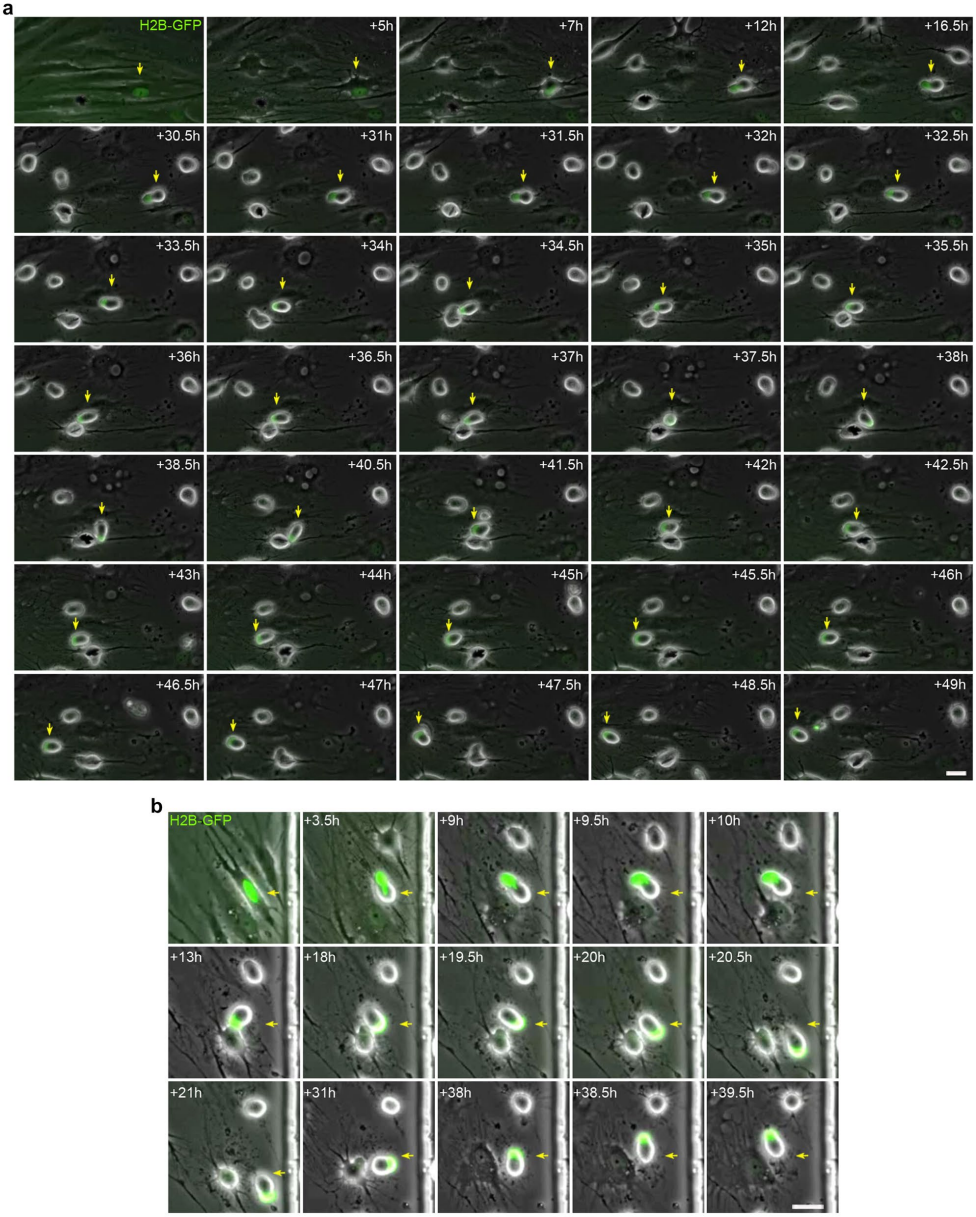
Mononucleated hBM-MSC-derived intermediate cells position their nucleus at the front of the cell during migration. (**a**) Time-lapse microscopy showed that kidney-shaped, histone H2B-GFP-expressing, hBM-MSC-derived intermediate cells positioned their nucleus at the front of the cell during migration. (**b**) In addition, finger-shaped, histone H2B-GFP-expressing, hBM-MSC-derived intermediate cells also positioned their nuclei at the front of the cell during migration. Scale bar: 25 µm.

**Figure 8:**
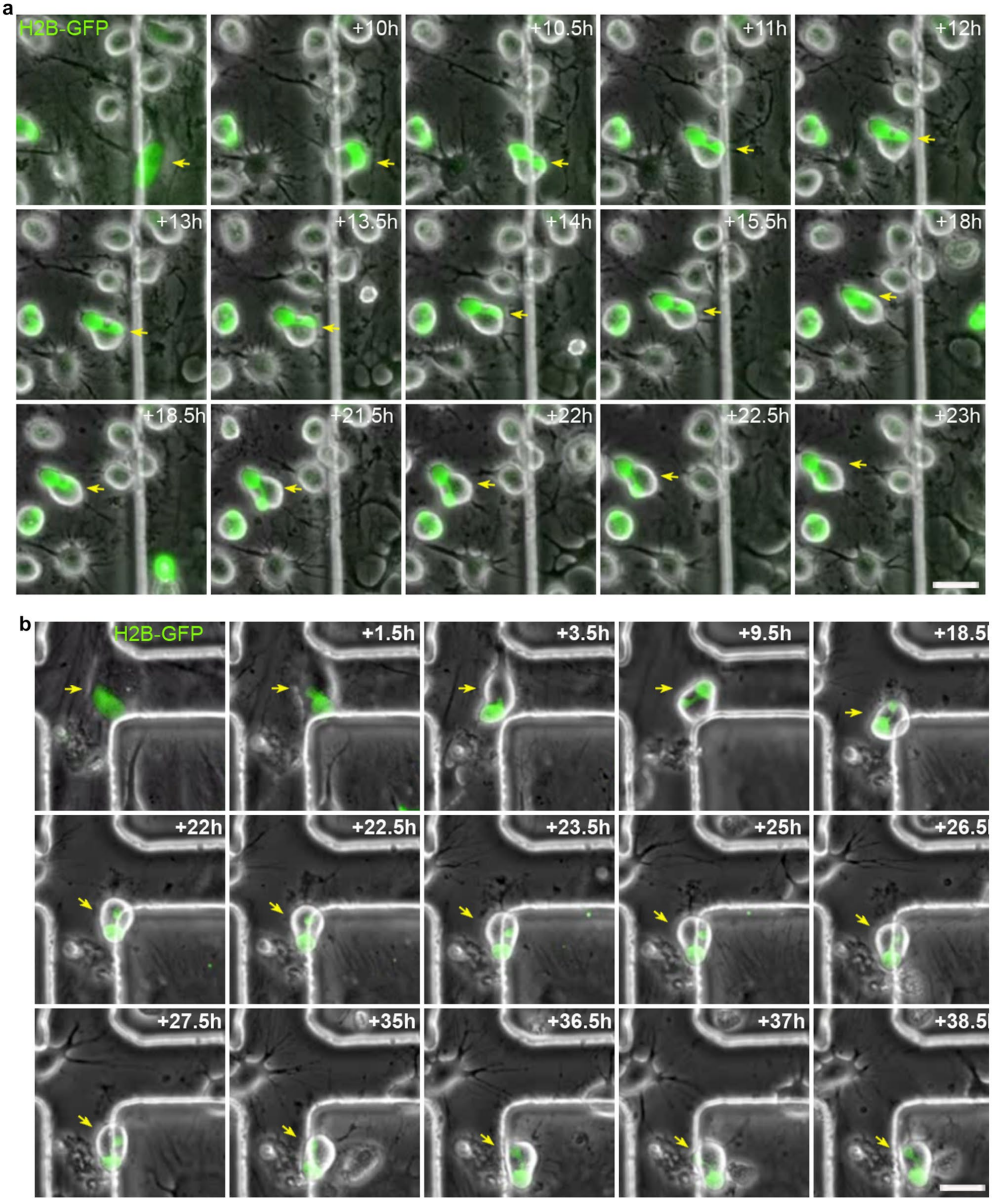
hBM-MSC-derived intermediate cells with lobed nuclei position their nucleus at the front of the cell during migration. (**a**) Time-lapse microscopy showed that histone H2B-GFP-expressing, hBM-MSC-derived intermediate cells with lobed nuclei positioned their nucleus at the front of the cell during migration. (**b**) Futhermore, histone H2B-GFP- expressing, hBM-MSC-derived intermediate cells with lobed nuclei positioned their largest lobe at the front of the cell during migration. Scale bar: 25 µm.

It is important to note that histone H2B-GFP-expressing, hBM-MSC-derived intermediate cells position their nucleus at the front of the cell during migration (Fig. 7 and Supplementary Videos S10 and S11). This nuclear positioning was observed in mononucleated hBM-MSC-derived intermediate cells, in both cells with kidney shaped nuclei (Fig. 7a and Supplementary Videos S10) and cells with finger shaped nuclei (Fig. 7b and Supplementary Video S11). hBM-MSC-derived intermediate cells with lobed nuclei also positioned their nucleus at the front of the cell during migration (Fig. 8a and Supplementary Video S12). Furthermore, we observed that hBM-MSC-derived intermediate cells with lobed nuclei positioned their largest lobe at the front of the cell during migration (Fig. 8b and Supplementary Video S13). These finding are consistent with a previous study that reported that human leukocytes position their nuclear lobes at the front of the cell during migration^36^.

As mentioned previously, hBM-MSC-derived intermediate cells can also assume new morphologies, gradually adopting a neural-like morphology through active neurite extension or re-differentiating back to their mesenchymal morphology. There were no major changes in nuclear positioning or lobed nuclei formation as histone H2B-GFP-expressing, hBM-MSC-derived intermediate cells gradually acquired a neural-like morphology (Fig. S6 and Supplementary Video S14). By contrast, when histone H2B-GFP-expressing, hBM-MSC-derived intermediate cells reverted back to the mesenchymal morphology, the nuclei from mononucleated intermediate cells gradually adopted their original ellipsoid shape (Fig. S7a and Supplementary Video S15). Yet when intermediate cells with lobed nuclei reverted back to the mesenchymal morphology, the lobed nuclei preserved their shape for hours (Fig. S7b and Supplementary Video S16). In future studies, live-cell nucleus fluorescence labelling and time-lapse microscopy over longer periods is necessary to determine whether the lobed nuclei finally fused to form a single nucleus.

Finally, laser scanning confocal microscopy revealed that many histone H2B-GFP- expressing, hBM-MSC-derived intermediate cells with unusual nuclear structures also exhibited extranuclear chromatin-containing bodies in the cellular cytoplasm during neural-like differentiation (Fig. S8). We observed that hBM-MSCs with finger shaped nuclei (Fig. S8a), kidney shaped nuclei (Fig. S8b) and lobed nuclei connected by nucleoplasmic bridges (Fig. S8c) can also exhibit extranuclear bodies in the cellular cytoplasm. Furthermore, we found chromatin-containing bodies connected to the main body of the nucleus by thin strands of nuclear material (Fig. S8d), chromatin-containing bodies moving away from or toward the main nuclei (Fig. S8e) and two lobed nuclei unconnected by any nucleoplasmic bridges with chromatin-containing bodies in the cellular cytoplasm (Fig. S8f). These results indicate that binucleated hBM-MSCs form during neural-like differentiation with independence of any cell division or fusion events.

Importantly, the nuclear morphology of hBM-MSCs observed during the neural-like differentiation bears a lot of similarities to the nuclear morphology of neural stem cells located in the ventricular-subventricular zone of the anterolateral ventricle wall of the human foetal brain^37^ and adult mouse brain^38–40^; their nuclear morphology is also very similar to that of many cultured hippocampal neurons^41^. For example, the nuclear morphologies in Fig. S8c are very similar to those observed in Wittmann et al.^41^ (their Fig. S2), Cebrián-Silla et al.^40^ (their Figs. 1E, 4I, 6F, S1B, S2B and S6A), Guerrero-Cázares et al.^37^ (their Fig. 2C) and Doetsch et al.^38^ (their Fig. 3A). Furthermore, the nuclear morphologies shown in Fig. S8d closely resemble those reported by Cebrián-Silla et al^40^ (their Figs. 1A,1B,1M,1N, 3I, 6A, 6B, 6H) and Guerrero-Cázares et al.^37^ (their Fig. 3j). In addition, the nuclear morphologies in Fig. S8e are very similar to those observed in Capilla-Gonzalez et al^39^ (their Fig. S2C). Although it has been suggested that these unusual nuclear structures are associated with quiescence in adult neural stem cells^40^, our results suggest that they are associated with nuclear movement within the cell during neuronal differentiation, but without any relation to cell division.

## Discussion

In this study, we have shown that when hBM-MSCs were exposed to a neural induction medium, they rapidly reshaped from a flat to a spherical morphology (hBM-MSC-derived intermediate cells). Subsequently, hBM-MSC-derived intermediate cells could preserve this the spherical morphology or assume new ones; they gradually adopted a neural-like morphology through active neurite extension or re-differentiated back to the mesenchymal fate. Furthermore, we found that hBM-MSCs can rapidly and repeatedly switch lineages without cell division. Importantly, there was no cell proliferation or cell fusion through neural-like differentiation from hBM-MSCs.

This work also highlights that nuclear remodelling occurred during in vitro neural-like differentiation from hBM-MSCs. We discovered that nuclei in intermediate cells rapidly moved within the cell, adopting different morphologies and even forming lobed nuclei. These nuclear movements generated transient cellular protrusions that appeared and disappeared from the surface of hBM-MSC-derived intermediate cells. The intermediate cells positioned their nucleus at the front of the cell during migration.

The literature published in recent decades has shown that MSCs isolated from different adult tissues can rapidly transdifferentiated into neural-like cells in culture^10–18^. However, the findings and their interpretation have been challenged. It has been argued that the rapid neural transdifferentiation of MSCs reported in culture studies is actually due to cytotoxic changes induced by the media^24,26–28^, so the rapid changes should not be interpreted as signs of transdifferentiation. In this study, we have shown that hBM-MSCs can rapidly adopt a neural-like morphology via active neurite extension. New neurites grew from the body of some intermediate cells, which gradually adopted a more complex morphology, by acquiring dendrite-like and axon-like domains. These result confirm our previous findings^33,34^ and provide a stronger basis for rejecting the idea that the rapid acquisition of a neural-like morphology during MSC transdifferentiation is merely an artefact. However, it is important to mention that future research is required to optimize the diverse array of in vitro neural induction protocols that have been devised for MSCs^42^ in order to achieve the goal of differentiating MSCs into functional neurons.

As mentioned previously, hBM-MSC-derived intermediate cells could also re-differentiated back to the mesenchymal fate. Furthermore, we found that hBM-MSCs can rapidly and repeatedly switch lineages without cell division. It has been claimed that the rapidity with which the neuron-like morphology is both gained and lost also argues againts physiological differentiation^43^. However, a recent study has demostrated that Schwann cells can also rapidly undergo multiple cycles of differentiation and dedifferentiation without entering the cell cycle^35^.

While transplantation studies indicated that BMDCs and MSCs can contribute to the neuronal architecture of the central nervous system, including that of Purkinje cells within the cerebellum^6–8,21–23^, it remains unclear whether the underlying mechanism is transdifferentiation or BMDC fusion with the existing neuronal cells, or both^25^. Cell fusion has been put forward to explain the presence of gene-marked binucleated Purkinje neurons after gene-marked bone marrow-derived cell transplantation^29,30^. Evidence supporting cell fusion derived from experiment using Cre/lox recombination to detect fusion events^29^. Bone marrow cells expressing both GFP and Cre recombinase where grafted in R26R mice. The study confirmed the presence of two independent nuclei in a LacZ-positive and GFP-negative Purkinje cell, but did not show any LacZ-positive and GFP-positive binucleated Purkinje cell. Therefore, Alvarez-Dolado et al. does not conclusively demonstrate that cell fusion is the underlying mechanism to explain the presence of binucleated Purkinje neurons after bone marrow-derived cell transplantation. Futher support to cell fusion came from experiment using GFP-positive sex-mis-matched bone marrow transplants in mice^30^. GFP-positive Purkinje cells where shown to contain two nuclei, with one of these nuclei containing the Y chromosome. This study does not conclusively demonstrate that GFP-positive Purkinje neurons found in the host cerebellum are the result of fusion between a host female Purkinje cell and a male bone marrow-derived cells, only demonstrates that binucleated Purkinje cells express markers of donor origin.

In this study, we demonstrated that binucleated hBM-MSCs can be formed during neural-like differentiation with independence of any cell fusion. Therefore, our results provide evidence that transdifferentiation may be also the mechanism behind the presence of gene-marked binucleated Purkinje neurons after gene-marked bone marrow-derived cell transplantation. It is important to note that binucleated Purkinje neurons are also present in healthy, unmanipulated mice^44^. Futhermore, human studies have also found binucleated Purkinje neurons in non-transplanted individuals^25^. What is more, many authors have reported binucleated neurons in various central and peripheral parts of the nervous system including, the cerebral cortex, cerebellum, sympathetic and spinal ganglia, diencephalon, midbrain, hindbrain, cerebral cortex and spinal cord^45–48^.

As mentioned previously, the nuclear morphology of hBM-MSCs observed during the neural-like differentiation bears a lot of similarities to the nuclear morphology of neural stem cells located in the ventricular-subventricular zone of the anterolateral ventricle wall of the human foetal brain^37^ and adult mouse brain^38–40^; their nuclear morphology is also very similar to that of many cultured hippocampal neurons^41^.

Although it has generally been believed that adult neurogenesis occurs progressively through sequential phases of proliferation and neuronal differentiation of adult stem cells^49^, the approaches used to probe stem cell division and differentiation, and even direct lineage tracing, are inherently limited^50–54^. These findings indicate that there is a lack of a reliable definitive method to label dividing progenitors and follow their progeny^54^. It is important to note that almost none of reports describing newborn neurons in the adult brain have showed mitotic figures^38,55–60^. Therefore, to date, there is no conclusive evidences to consider that adult neurogenesis occurs progressively through sequential phases of proliferation and neuronal differentiation of adult stem cells. Collectively, these findings suggest the possibility that new neurons can also be generated in the adult brain without necessitating cell division. Future research is required to determine the likelihood of this premise.

The most important discovery in this work is the observation that nuclei in intermediate cells rapidly move within the cell, adopting different morphologies and even forming binucleated cells. This is the first direct evidence, to our knowledge, that nuclei of human cells can change position and morphology so quickly. We noted that nuclear movements generated transient cellular protrusions that appeared and disappeared from the surface of intermediate cells. Futhermore, the intermediate cells positioned their nucleus at the front of the cell during migration. These findings may suggest that nuclei in intermediate cells are somehow sensing their surroundings. Future research is required to determine the feasibility of this conjecture.

This study not only shows that the main nuclei move within the cell, changing their morphology and position, but also that there are different sized chromatin-containing extranuclear bodies within cell cytoplasm. These DNA containing structures displayed a spherical or ovoid shape^33,34^. It is important to note that a recent study has demonstrated that chromatin-containing bodies arise from the main nuclei, move within the cell and are ultimately loaded in exosomes^61^. Therefore, it would be also interesting to examine whether chromatin-containing bodies can move independently of the movement of the main nuclei or if they are a product of the main nuclei movement.

Beyond the central nervous system, the presence of lobed nuclei has been reported in most immune cells, but the functional significance of multilobed nuclear structures is not yet known^62,63^. What is known is that human leukocytes position their nuclear lobes at the front of the cell during migration^36^. Importantly, the intermediate hBM-MSCs also positioned their nucleus at the front of the cell during migration. Futhermore, binuclear cells are commonly found in various human organs including, heart and liver. Cardiomyocytes possess a unique ability to transition from mononucleate to the mature binucleate phenotype in late fetal development and around birth^64^. Hepatocytes commonly have two nuclei were often linked by connecting DNA strands which span the gap between the two nuclei^65^. The functional significance of binuclear cardiomyocytes and hepatocytes is also not yet known^64,65^. It would be interesting to examine whether the nuclear structures observed in immune cells, hepatocytes and cardiomyocytes are also associated with nuclear movement within the cell.

In the classical view of cell development, progenitor cells differentiate into several distinct cell intermediates, with an increasingly restricted lineage potential, until the final mature cell types are generated. The differentiated state of a cell was believed to be terminal and irreversible^1^. While asymmetric cell division is considered to be the mechanism by which the asymmetric inheritance of cellular components during mitosis defines the distinct fate of each daughter cell. However, there is increasing evidence that these rules can be broken. It has long been accepted that adult cells can assume new fates without asymmetric cell division through dedifferentiation and transdifferentiation processes, a phenomenon known as cellular plasticity^2–5^. Current research aims to understand the mechanisms of these these cell conversion processes and eventually harness them for use in regenerative medicine^66^. Our results provide additional evidence that adult cells can assume new fates without asymmetric cell division and lend further support to the notion that MSCs transdifferentiate towards a neural lineage through a dedifferentiation step followed by re-differentiation to neural phenotypes. Mesenchymal stromal cells could help increase our understanding of the mechanisms underlying cellular plasticity.

The actual occurrence of neuronal transdifferentiation of MSCs is currently much debated, but would have immense clinical potential in cell replacement therapy of neurodegenerative diseases^15^. We believe that, to date, there is no conclusive evidence to consider that MSC neural transdifferentiation is merely an artefact.

## Methods

### Ethical conduct of research

The authors declare that all protocols used to obtain and process all human samples were approved by the local ethics committees of the Miguel Hernández University of Elche (No. UMH.IN.SM.03.16) and University of Murcia (HULP3617.05/07/2012; HUSA19/1531.17/02/2020) according to Spanish and European legislation and conformed to the ethical guidelines of the Helsinki Declaration. Donors provided written informed consent before obtaining samples.

### Isolation and culture of hBM-MSCs

Bone marrow aspirates were obtained by percutaneous direct aspiration from the iliac crest of 5 healthy volunteers at University Hospital Virgen de la Arrixaca (Murcia, Spain). Bone marrow was collected with 20 U/ml sodium heparin, followed by a Ficoll density gradient-based separation by centrifugation at 540 g for 20 min. After, mononuclear cell fraction was collected, washed twice with Ca^2+^/Mg^2+^-free phosphate buffered saline (PBS) (Gibco Invitrogen) and seeded into 175-cm2 culture flasks (Nunc, Thermo Fisher Scientific) at a cell density 1.5 × 10^5^ cells/cm^2^ in serum-containing media, composed of DMEM low glucose medium (Thermo Fisher Scientific) supplemented with 10% fetal bovine serum (FBS; Lonza), 1% GlutaMAX (Thermo Fisher Scientific), non-essential amino acid solution (Sigma-Aldrich) and 1% penicillin/streptomycin (Thermo Fisher Scientific). After 3 days of culture at 37 °C and 7% CO_2_, non-attached cells were removed and fresh complete medium was added. Culture media were renewed every 2 days, and the isolated hBMSCs were passaged when cultures were 70–80% confluent. All studies were performed using hBMSCs expanded within culture passages 3–4.

### Expression vectors and cell transfection

The expression vectors used in the present study were H2B-eGFP, a gift from Geoff Wahl (Addgene plasmid # 11680; http://n2t.net/addgene:11680; RRID:Addgene_11680; Kanda et al.^67^). Isolated hBMSCs-derived cells were transfected using the Gene Pulser-II Electroporation System (Bio-Rad Laboratories). Electroporation was performed in a sterile cuvette with a 0.4-cm electrode gap (Bio-Rad Laboratories), using a single pulse of 270 V, 500 μF. Plasmid DNA (5 μg) was added to 1.5 × 10^6^ viable hBMSCs-derived cells in 0.2-ml DMEM low glucose medium (Thermo Fisher Scientific) before electrical pulsing. hBM-MSCs-derived cells were transfected 1 day before neural differentiation.

### Time-lapse microscopy of histone H2B-GFP expressing hBM-MSCs cultured in neural induction media

We used μ-Dish 35 mm, high Grid-500 (Ibidi) for live cell imaging. Histone H2B-GFP transfected hBM-MSCs were plated onto collagen IV (Sigma-Aldrich) coated plastic or glass coverslips. To induce neural differentiation, cells at passage 3–4 were allowed to adhere to the plates overnight. Basal media was removed the following day and the cells were cultured for 2 days in serum-free media (designated as the neural basal media) consisting in Dulbecco’s modified Eagle’s medium/F12 (DMEM/F12 Glutamax, Gibco) supplemented with N2-supplement (R&D systems), 0.6% glucose (Sigma-Aldrich), 5 mM HEPES (Sigma-Aldrich), 0.5% human serum albumin (Sigma-Aldrich), 0.0002% heparin (Sigma-Aldrich), non-essential amino acid solution (Sigma-Aldrich) and 100 U/ml penicillin–streptomycin (Sigma-Aldrich). On day 3, cells were cultured in neural induction media, consisting in the neural basal media supplemented with 500 nM retinoic acid (Sigma-Aldrich), 1 mM dibutyryl cAMP (Sigma-Aldrich) and the growth factors BDNF (10 ng/ml; Peprotech), GDNF (10 ng/ml; Peprotech) and IGF-1 (10 ng/ml; R&D systems). Time-lapse analysis was carried out using a Widefield Leica Thunder-TIRF imager microscope. We perform time-lapse microscopy within the first 71 h after neural induction media was added directly to the cells. Time-lapse images were obtained every 30 min. During imaging, cells were enclosed in a chamber maintained at 37 °C under a humidified atmosphere of 5% CO_2_ in air. Data are representative of ten independent experiments.

### Immunocytochemistry

A standard immunocytochemical protocol was used as previously described (Bueno et al.^32–34^). Histone H2B-GFP transfected hBM-MSCs were plated onto collagen IV (Sigma-Aldrich) coated plastic or glass coverslips and maintained in neural induction media. Cells were rinsed with PBS and fixed in freshly prepared 4% paraformaldehyde (PFA; Sigma-Aldrich). Fixed cells were blocked for 2 h in PBS containing 10% normal horse serum (Gibco) and 0.25% Triton X-100 (Sigma) and incubated overnight at 4 °C with antibodies against β-III-tubulin (TUJ1; 1:500, Covance) in PBS containing 1% normal horse serum and 0.25% Triton X-100. On the next day, cells were rinsed and incubated with the secondary antibody conjugated with Alexa Fluor® 594 (anti-mouse; 1:500, Molecular Probes). Cell nuclei were counterstained with DAPI (0.2 mg/ml in PBS, Molecular Probes).

### Mesenchymal stromal cell immunophenotyping

Human BM-MSCs were characterized by flow cytometry. Three different pools of hBM-MSCs were analyzed the same day, and at least 5 × 10^4^ events for all samples were recorded. Briefly, cells were detached with TrypLE Express dissociation reagent (Gibco), washed, and resuspended in PBS (Gibco) containing 1% fetal bovine serum (Lonza). Then, hBM-MSCs were incubated with fluorochrome-conjugated anti-mouse antibodies specific for the surface markers CD73, CD90, CD105, CD14, CD20, CD34 and CD45 (all from BioLegend), or their specific control isotype antibodies, for 30 min in the dark at 4 °C. Finally, cells were washed, resuspended at 1 × 10^6^ cells/ml and analyzed in a FACSCanto II flow cytometer (BD Biosciences).

### Images and data analyses

Photograph of visible and fluorescent stained samples were carried out in a Widefield Leica Thunder-TIRF imager microscope equipped with a digital camera or in confocal laser scanning microscope Leica TCS-SP8. We used Filmora Video Editor software for video editing and Photoshop software to improve the visibility of fluorescence images without altering the underlying data.

## Supplementary Information

Supplementary Figures.

Supplementary Legends.

Supplementary Video S1.

Supplementary Video S2.

Supplementary Video S3.

Supplementary Video S4.

Supplementary Video S5.

Supplementary Video S6.

Supplementary Video S7.

Supplementary Video S8.

Supplementary Video S9.

Supplementary Video S10.

Supplementary Video S11.

Supplementary Video S12.

Supplementary Video S13.

Supplementary Video S14.

Supplementary Video S15.

Supplementary Video S16.

## Data Availability

All data generated or analysed during this study are included in this published article (and its supplementary information files).
