# Supplementary material for: Binucleated human bone marrow-derived mesenchymal cells can be formed during neural-like differentiation with independence of any cell fusion events
Source: Sci Rep. 2022 Nov 30;12:20615. doi: 10.1038/s41598-022-24996-8 (PMC9712539; doi:10.1038/s41598-022-24996-8)
Supplement: Supplementary file 1 — Supplementary Figures. [file 41598_2022_24996_MOESM1_ESM.docx]

**Supplementary information**

Binucleated human bone marrow-derived mesenchymal cells can be formed during neural-like differentiation with independence of any cell fusion events.

Authors: Carlos Bueno^1,*^, Miguel Blanquer^1^, David García-Bernal^1,2^, Salvador Martínez^3^ and José M. Moraleda^1^.


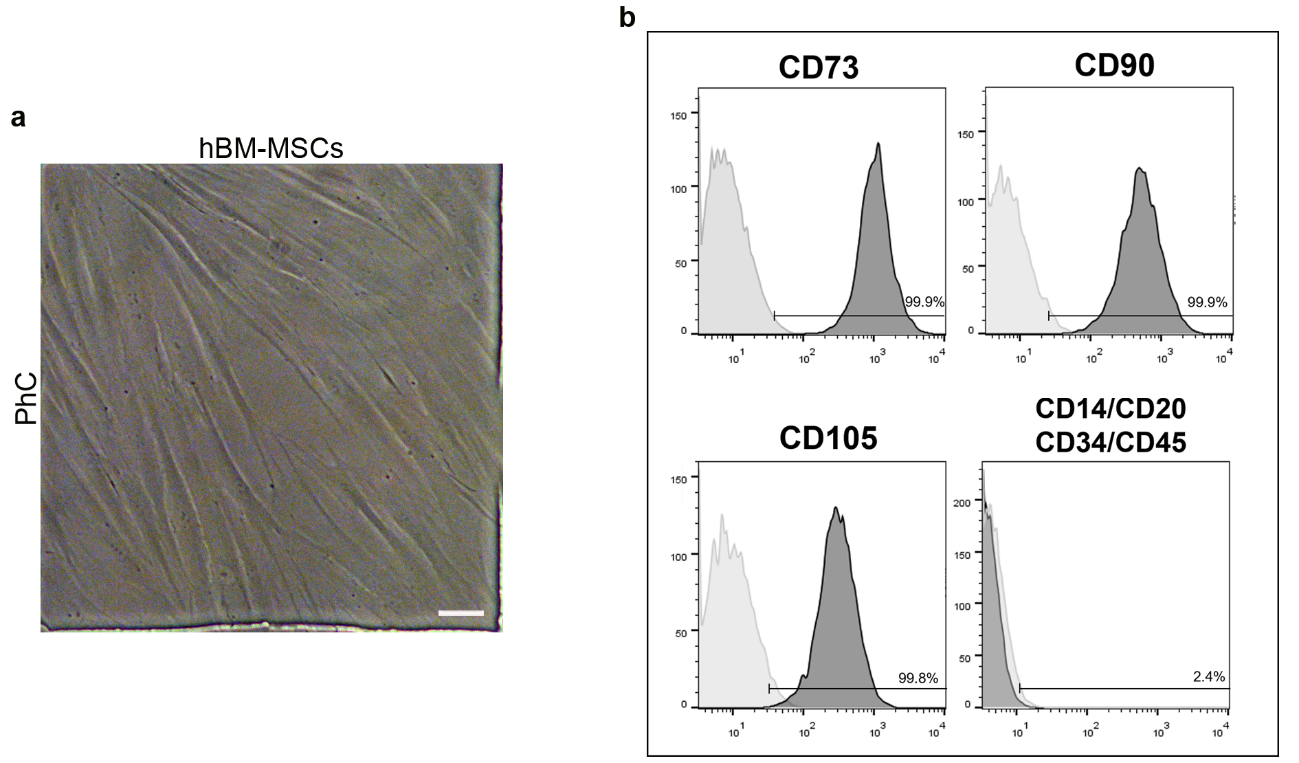


**Figure S1. Morphology and immunophenotypes of hBM-MSCs**. **a**) Under proliferation conditions, hBM-MSCs displayed a flat, fibroblast-like morphology with little evidence of refractility. Scale bar: 50 μm. PhC**:** Phase-contrast photomicrograft. **b**) MSC isolated from human bone marrow express typical MSC markers such as CD73, CD90 and CD105, whereas expression of the hematopoietic markers CD14, CD20, CD34, and CD45 are low or negative. Control isotype antibodies staining are also shown as light grey histograms. Histograms show representative flow cytometry results obtained from n=3 hBM-MSC cultures.


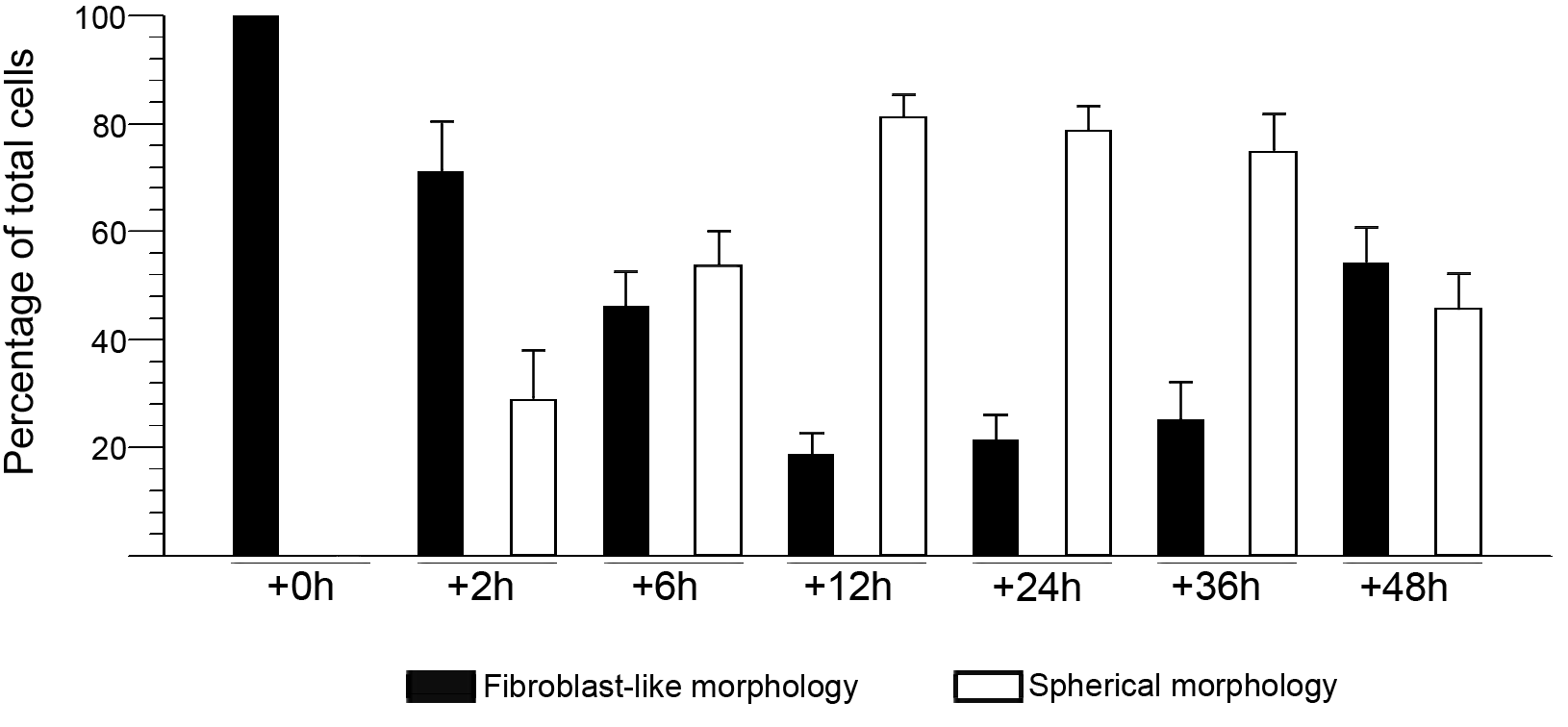


**Figure S2. The mayority of hBM-MSCs assumed a spherical morphology within 12 hours of exposure to neural induction media**. Percentage of cells showing fibroblast-like morphology and spherical shape over a time period of 48 hours after adding the neural induction medium. Results are the mean of ten independent experiments ± SD.


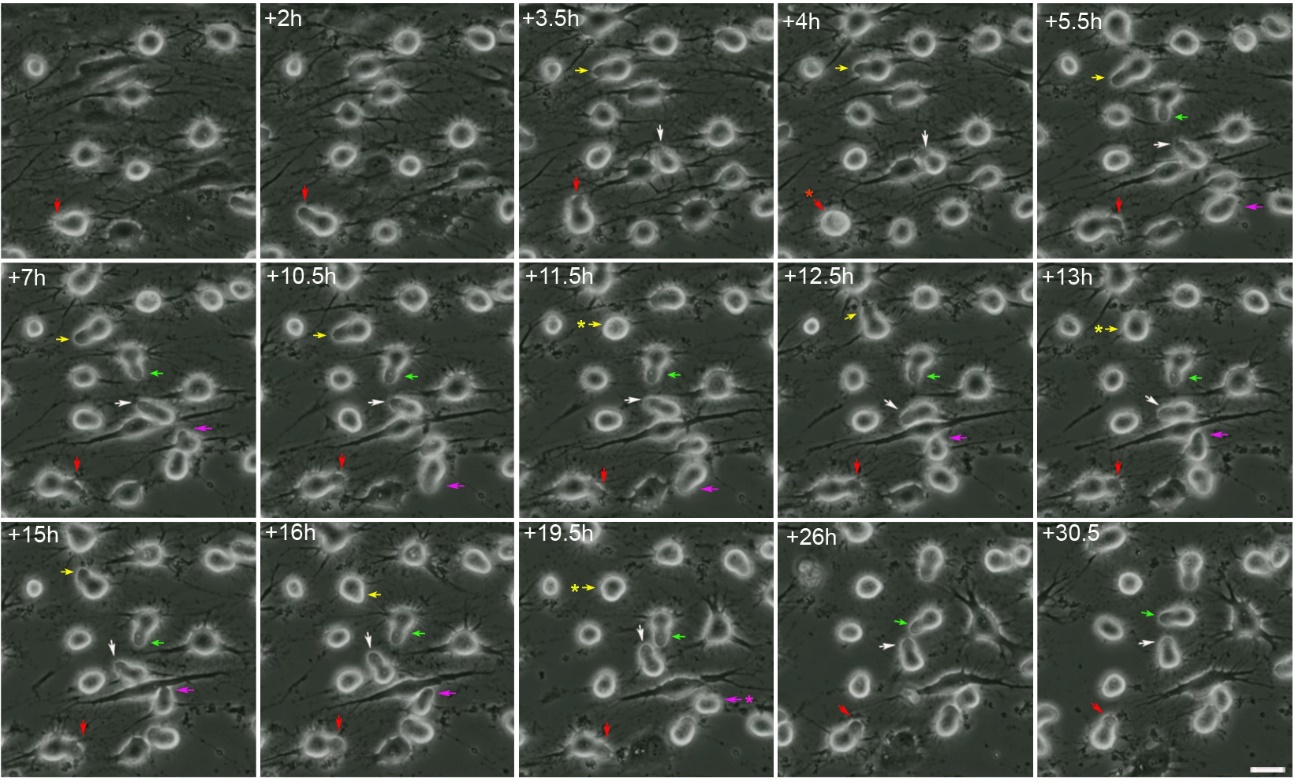


**Figure S3. Morphological changes in hBM-MSC-derived intermediate cells**. Time-lapse imaging showed the appearance (arrows), movement and disappearance (asterisk) of cellular protrusion from the surface of hBM-MSC-derived intermediate cells. Scale bar: 25 μm.


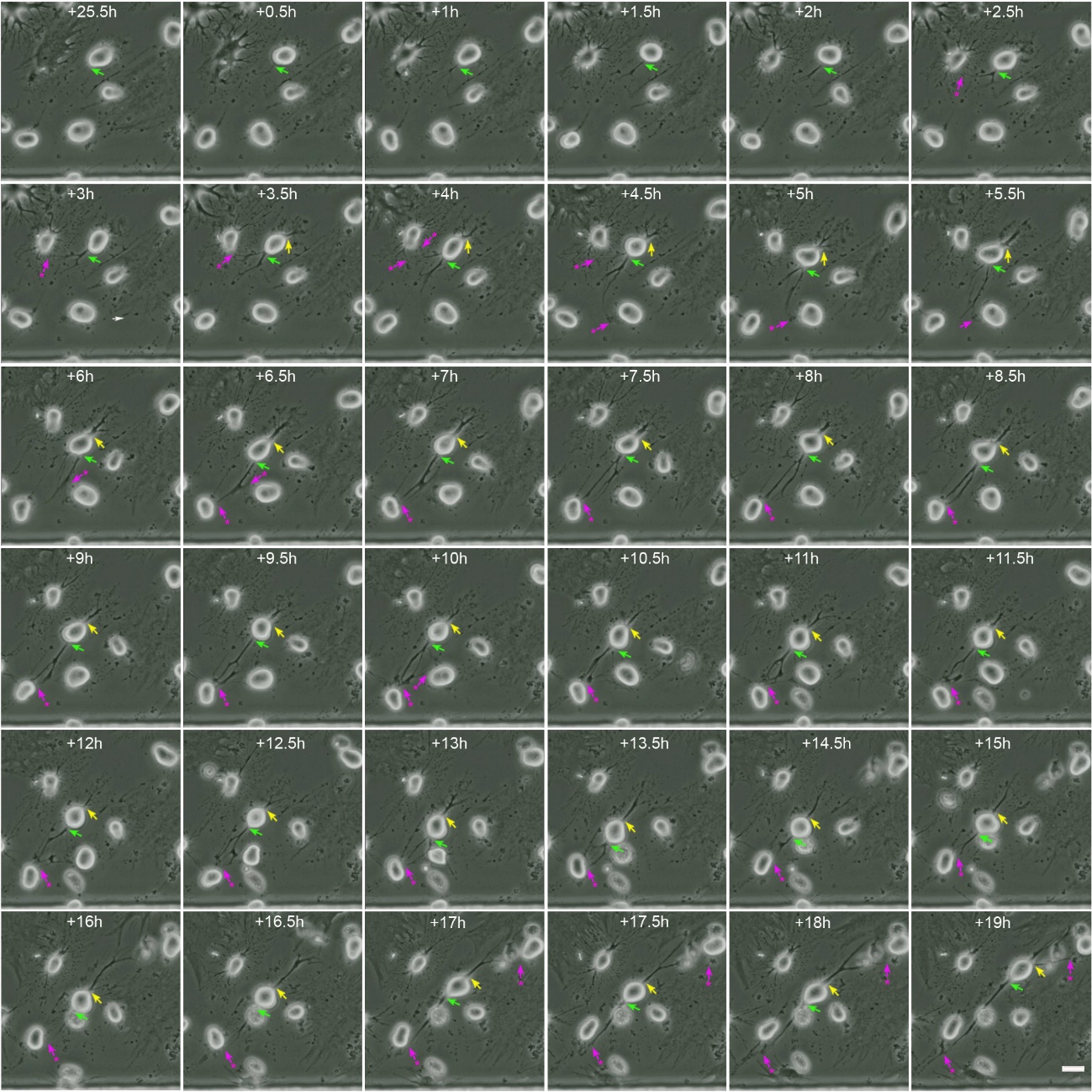


**Figure S4. hBM-MSC-derived neural-like cells create connections via extensions of their neurites**. Time-lapse imaging revealed the growth of new neurites from the body of round cells (intermediate cells) that which gradually adopted a complex morphology, acquiring dendrite-like (green arrows) and axon-like domains (yellow arrows). Time-lapse imaging also revealed that the hBM-MSC-derived neural-like cells create connections (yellow arrows) via extensions of their neurites. Scale bar: 25 μm.

**
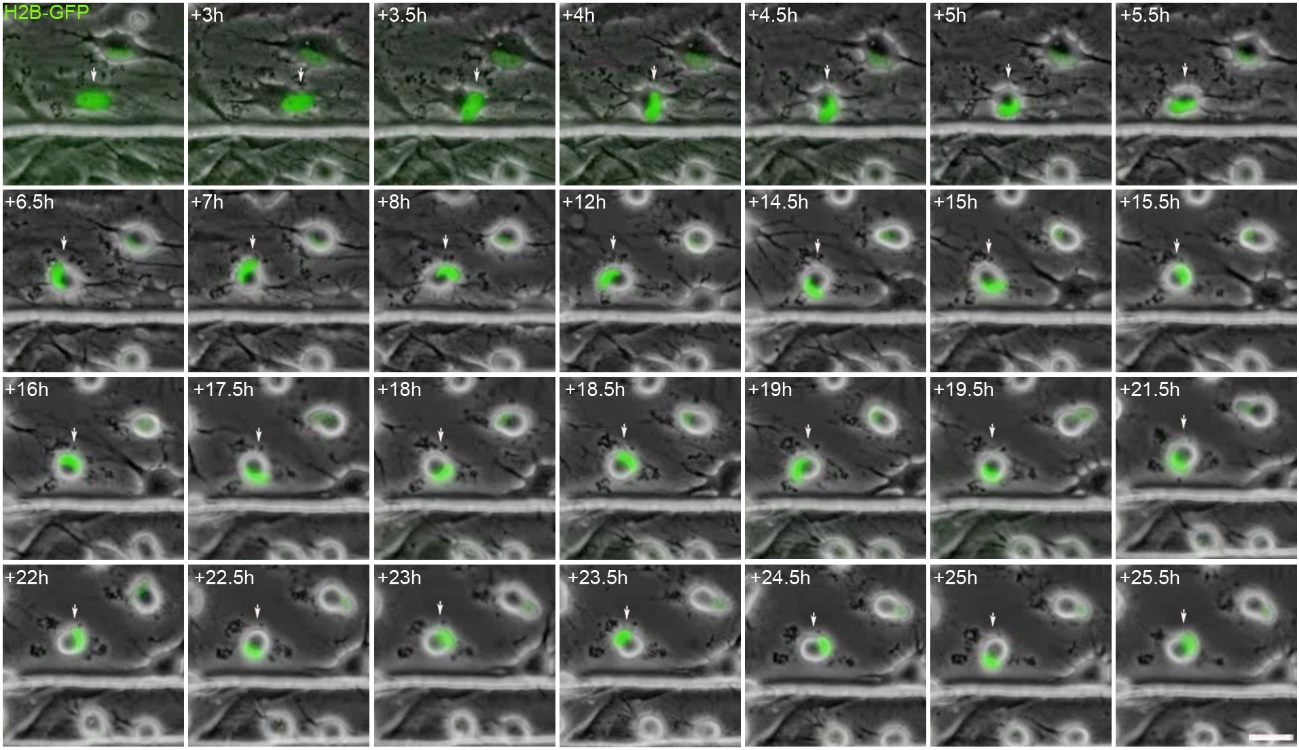
**

**Figure S5. Intermediate hBM-MSC nuclei can** **switch their morphology and positioning**. Time-lapse microscopy highlighted that the cell nucleus from histone H2B-GFP-expressing hBM-MSC-derived intermediate cells can switch its morphology while it is moving. Here, the nucleus acquired a finger-like shape before reorienting toward a peripheral position within the cell and acquiring a kidney-like shape. Subsequently, the cell nucleus began to move rapidly around the cell. Scale bar: 25 μm.

**
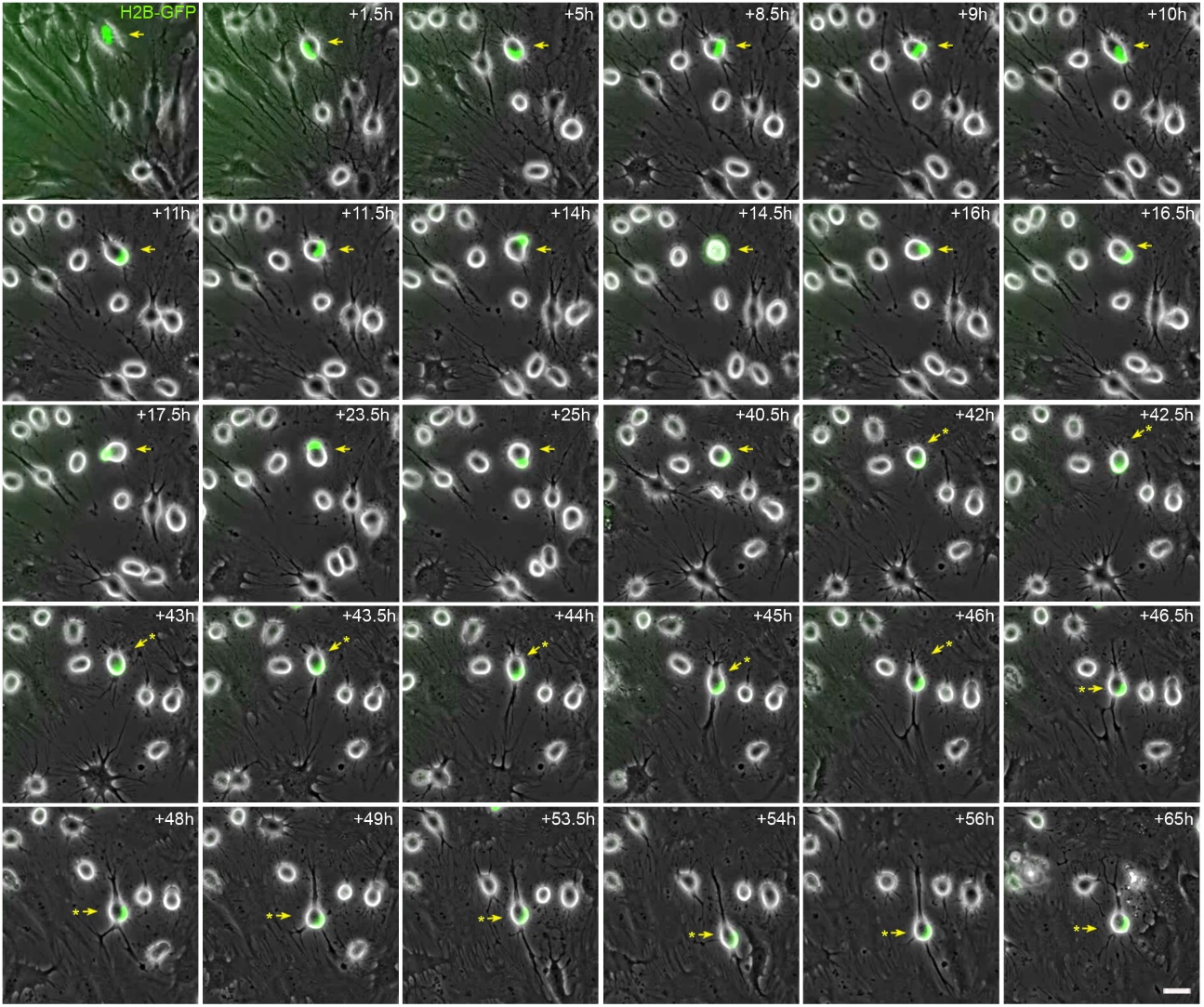
****Figure S6. Nuclear morphology and positioning during neuronal-like polarisation of hBM-MSC-derived intermediate cells**. Time-lapse microscopy did not reveal major changes in nuclear positioning or lobed nuclei formation as histone H2B-GFP-expressing, hBM-MSC-derived intermediate cells gradually acquired a neural-like morphology (asterisks). Scale bar: 25 μm.

**
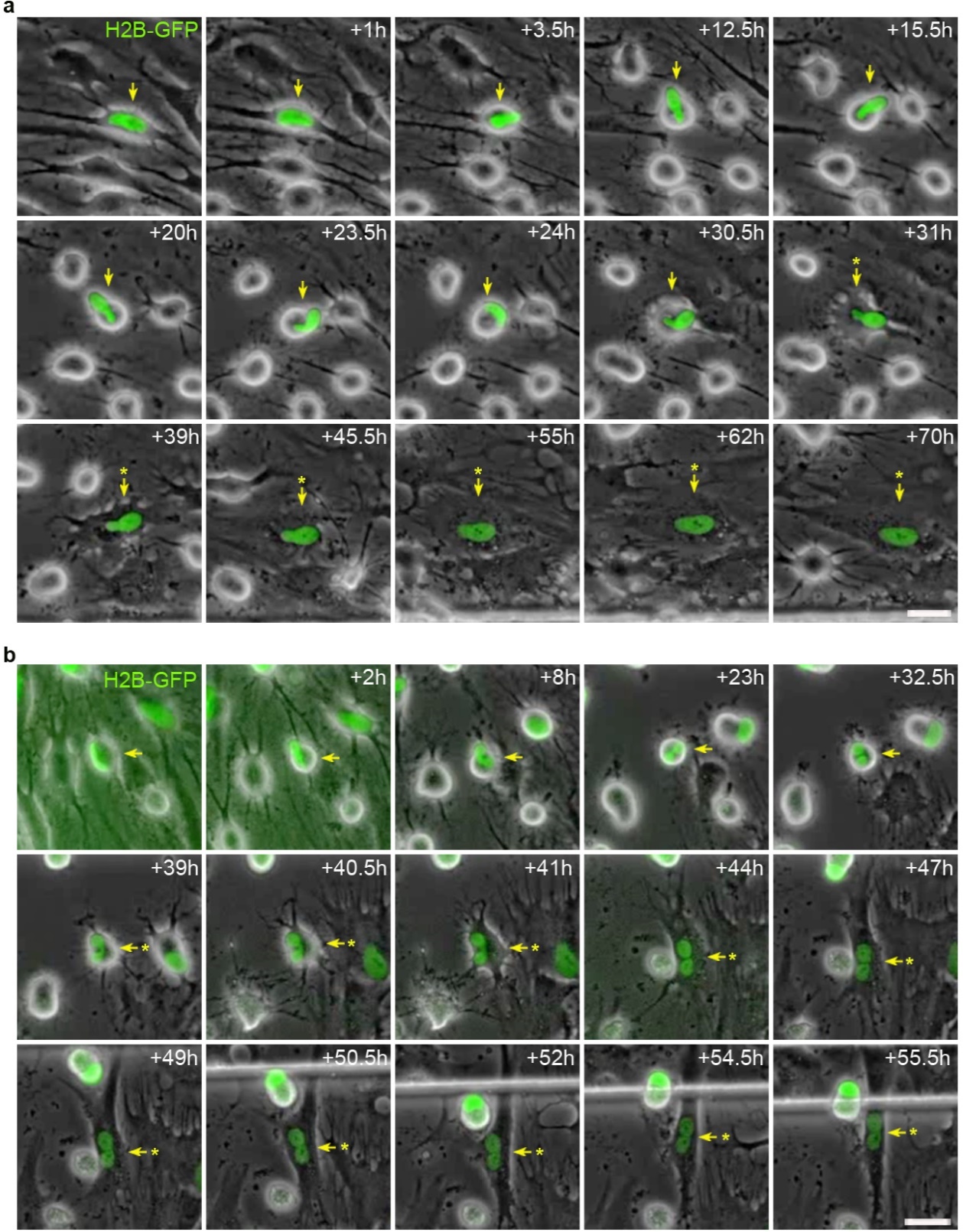
**

**Figure S7. Nuclear morphology when hBM-MSC-derived intermediate cells redifferentiate back to the mesenchymal fate**. **a**) Time-lapse microscopy revealed that when histone H2B-GFP-expressing, intermediate hBM-MSCs with a single nucleus reverted back to the mesenchymal morphology (asterisk), the nuclei gradually reverted back to their original ellipsoid shape. **b**) By contrast, when histone H2B-GFP-expressing, intermediate hBM-MSCs with lobed nuclei reverted back to the mesenchymal morphology (asterisk), the lobed nuclei were maintained for hours. Scale bar: 25 μm.

**
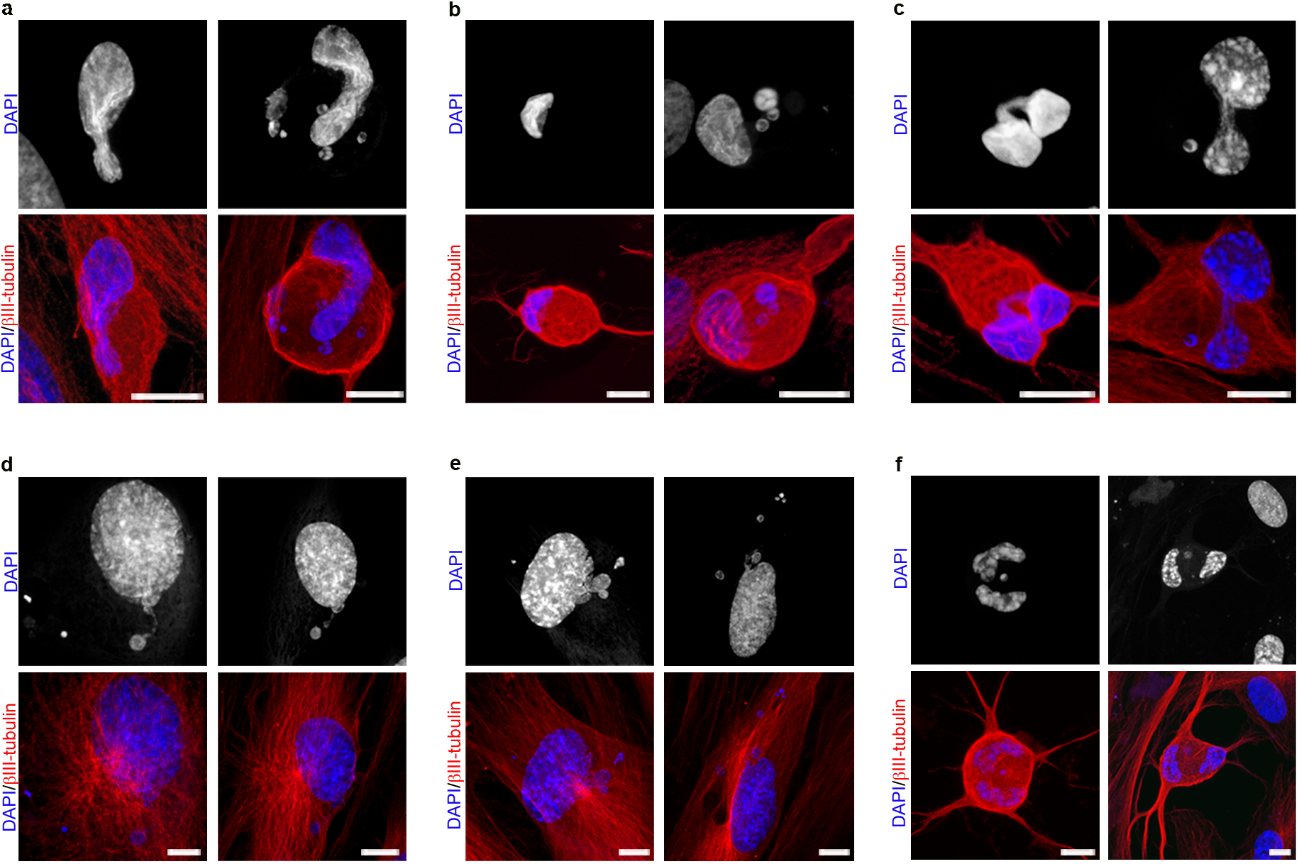
**

**Figure S8. hBM-MSCs exhibit unusual nuclear structures and chromatin-containing bodies in the cellular cytoplasm during neural-like differentiation**. **a**) Confocal microscopy analysis showed that histone H2B-GFP-expressing hBM-MSCs with a finger-shaped nucleus can also present chromatin-containing bodies in the cellular cytoplasm. **b**) Histone H2B-GFP-expressing hBM-MSCs a kidney-shaped nucleus can also exhibit chromatin-containing bodies in the cellular cytoplasm. **c**) Histone H2B-GFP-expressing hBM-MSCs with a lobed nucleus connected by nucleoplasmic bridges can also exhibit chromatin-containing bodies in the cellular cytoplasm. **d**) We noted that histone H2B-GFP-expressing hBM-MSCs with chromatin-containing bodies were connected to the main body of the nucleus by thin strands of nuclear material. **e**) Furthermore, histone H2B-GFP-expressing hBM-MSCs with chromatin-containing bodies moved away from or toward the main nuclei. **f**) Histone H2B-GFP-expressing hBM-MSCs with two lobed nuclei unconnected by any nucleoplasmic bridges with chromatin-containing bodies in the cellular cytoplasmwere also observed. Scale bar: 10 μm.
