# Supplementary material for: Binucleated human bone marrow-derived mesenchymal cells can be formed during neural-like differentiation with independence of any cell fusion events
Source: Sci Rep. 2022 Nov 30;12:20615. doi: 10.1038/s41598-022-24996-8 (PMC9712539; doi:10.1038/s41598-022-24996-8)
Supplement: Supplementary file 2 — Supplementary Legends. [file 41598_2022_24996_MOESM2_ESM.docx]

**Supplementary Video legends.**

**Supplementary Video S1.** **Morphological changes in hBM-MSC cultures during neural-like differentiation.** Time-lapse imaging revealed that, following neural induction, hBM-MSCs rapidly reshaped from a flat to a spherical morphology (hBM-MSC-derived intermediate cells). Subsequently, we observed that hBM-MSC-derived intermediate cells can preserve their spherical shape for several days, change to that of neural-like cells through active neurite extension or revert back to the mesenchymal morphology.

**Supplementary Video S2.** **Morphological changes in hBM-MSC-derived intermediate cells.** Time-lapse imaging showed the appearance, movement and disappearance of cellular protrusion from the surface of hBM-MSC-derived intermediate cells.

**Supplementary Video S3.** **Neuronal-like polarisation of hBM-MSC-derived intermediate cells.** Time-lapse imaging revealed the growth of new neurites from the body of intermediate cells that which gradually adopted a complex morphology, acquiring dendrite-like and axon-like domains. There was no observation of major transient cellular protrusion as hBM-MSC-derived intermediate cells gradually acquired a neural-like morphology.

**Supplementary Video S4. hBM-MSC-derived neural-like cells create connections via extensions of their neurites.** Time-lapse imaging revealed the growth of new neurites from the body of round cells (intermediate cells) that which gradually adopted a complex morphology, acquiring dendrite-like and axon-like domains. Time-lapse imaging also revealed that the hBM-MSC-derived neural-like cells create connections via extensions of their neurites.

**Supplementary Video S5.** **hBMSCs can repeatedly switch lineages.** Time-lapse imaging showed that hBM-MSCs can also rapidly switch lineages without cell division.

**Supplementary Video S6.** **Nucleus remodelling occurs during neural-like differentiation from hBM-MSCs**. Time-lapse microscopy evidenced that nuclear remodelling occurred during neural-like differentiation from histone H2B-GFP-expressing hBM-MSCs. Nuclei from hBM-MSC-derived intermediate cells moved within the cell, adopting different morphologies, including finger shaped and kidney shaped, and even forming lobed nuclei connected by nucleoplasmic bridges.

**Supplementary Video S7.** **Nuclear movement generated cellular protrusions that appeared and disappeared from the surface of hBM-MSC-derived intermediate cells.** Time-lapse microscopy revealed that the cell nucleus of histone H2B-GFP-expressing hBM-MSCs acquired a finger-like shape and moved within the cell, generating the transient cellular protrusions on the surface of hBM-MSC-derived intermediate cells.

**Supplementary Video S8.** **Intermediate hBM-MSC nuclei can** **switch their morphology and positioning.** Time-lapse microscopy highlighted that the cell nucleus from histone H2B-GFP-expressing hBM-MSC-derived intermediate cells can switch its morphology while it is moving. Here, the nucleus acquired a finger-like shape before reorienting toward a peripheral position within the cell and acquiring a kidney-like shape. Subsequently, the cell nucleus began to move rapidly around the cell.

**Supplementary Video S9.** **Binucleated hBM-MSCs can form with independence of any cell fusion events.** Time-lapse microscopy revealed that the nuclei from histone H2B-GFP- expressing, intermediate hBM-MSCs can move within the cell, forming lobed nuclei connected by nucleoplasmic bridges. The movement of the lobed nuclei also generated cellular transient protrusions from the surface of hBM-MSC-derived intermediate cells.

**Supplementary Video S10.** **kidney-shaped intermediate hBM-MSCs cells positioned their nucleus at the front of the cell during migration.** Time-lapse microscopy showed that kidney-shaped, histone H2B-GFP-expressing, intermediate hBM-MSCs cells positioned their nucleus at the front of the cell during migration.

**Supplementary Video S11.** **Finger-shaped intermediate hBM-MSCs positioned their nucleus at the front of the cell during migration.** Time-lapse microscopy showed that finger-shaped histone H2B-GFP-expressing, intermediate hBM-MSCs also positioned their nuclei at the front of the cell during migration.

**Supplementary Video S12.** **hBM-MSC-derived intermediate cells with lobed nuclei position their nucleus at the front of the cell during migration**. Time-lapse microscopy showed that histone H2B-GFP-expressing, intermediate hBM-MSCs with lobed nuclei positioned their nucleus at the front of the cell during migration.

**Supplementary Video S13.** **hBM-MSC-derived intermediate cells with lobed nuclei position their largest lobe at the front of the cell during migration** Time-lapse microscopy showed that histone H2B-GFP-expressing, intermediate hBM-MSCs with lobed nuclei positioned their largest lobe at the front of the cell during migration.

**Supplementary Video S14.** **Nuclear morphology and positioning during neuronal-like polarisation of hBM-MSC-derived intermediate cells.** Time-lapse microscopy did not reveal major changes in nuclear positioning or lobed nuclei formation as histone H2B-GFP-expressing, hBM-MSC-derived intermediate cells gradually acquired a neural-like morphology.

**Supplementary Video S15.** **Nuclear morphology when hBM-MSC-derived mononucleated intermediate cells redifferentiate back to the mesenchymal fate** Time-lapse microscopy revealed that when histone H2B-GFP-expressing, intermediate hBM-MSCs with a single nucleus reverted back to the mesenchymal morphology, the nuclei gradually reverted back to their original ellipsoid shape.

**Supplementary Video S16.** **Nuclear morphology when hBM-MSC-derived binucleated intermediate cells redifferentiate back to the mesenchymal fate**. Time-lapse microscopy revealed that when histone H2B-GFP-expressing, intermediate hBM-MSCs with lobed nuclei reverted back to the mesenchymal morphology, the lobed nuclei were maintained for hours.
